# Targeted deletion of the TSLP receptor reveals cellular mechanisms that promote type 2 airway inflammation

**DOI:** 10.1038/s41385-020-0266-x

**Published:** 2020-02-17

**Authors:** Hiroki Kabata, Anne-Laure Flamar, Tanel Mahlakõiv, Saya Moriyama, Hans-Reimer Rodewald, Steven F. Ziegler, David Artis

**Affiliations:** 1 Jill Roberts Institute for Research in Inflammatory Bowel Disease, Friedman Center for Nutrition and Inflammation, Joan and Sanford I. Weill Department of Medicine, Department of Microbiology and Immunology, Weill Cornell Medicine, Cornell University, New York, NY 10021 USA; 2 Division of Cellular Immunology, German Cancer Research Center (DKFZ), Heidelberg, 69120 Germany; 3 Benaroya Research Institute, Immunology Research Program, Seattle, Washington, 98101 USA; 4 Present Address: Division of Pulmonary Medicine, Department of Medicine, Keio University School of Medicine, Shinjuku, Tokyo, 160-8582 Japan; 5 Present Address: Department of Immunology, National Institute of Infectious Diseases, Shinjuku, Tokyo, 162-8640 Japan

## Abstract

Thymic stromal lymphopoietin (TSLP) is a critical upstream cytokine inducing type 2 inflammation in various diseases, including asthma and atopic dermatitis. Accumulating evidence suggests that TSLP can directly stimulate a variety of immune cells, such as dendritic cells (DCs), basophils, T cells, and group 2 innate lymphoid cells (ILC2s). However, which cell types directly respond to TSLP in vivo and how TSLP initiates type 2 inflammation has remained controversial. To define the precise role of TSLP in vivo, for the first time we generated multiple cell lineage-specific TSLP receptor-deficient mice to systematically dissect the cell-intrinsic requirements for TSLP responsiveness in type 2 inflammation in the lung. In papain-induced innate immune-mediated type 2 airway inflammation, TSLP directly stimulated ILC2s, but not basophils, leading to enhanced type 2 inflammation. On the other hand, in OVA-induced adaptive immune-mediated type 2 airway inflammation, TSLP principally acted on DCs and CD4 + T cells during the sensitization phase, but not basophils or ILC2s, and facilitated the development of Th2 cell-mediated airway inflammation. Together, these findings reveal that TSLP activates distinct immune cell cascades in the context of innate and adaptive immune-mediated type 2 inflammation.

## Introduction

Thymic stromal lymphopoietin (TSLP) is an IL-7-like cytokine produced by epithelial cells, keratinocytes, fibroblasts, and stromal cells in the context of infection and inflammation.^1^ Numerous studies have implicated TSLP in allergic diseases, including genome-wide association studies, which found that single nucleotide polymorphisms in *TSLP* locus were associated with an increased risk of asthma and atopic dermatitis.^2–5^ Moreover, the expression level of TSLP in the airway of asthmatic patients and the skin of atopic dermatitis patients was highly increased.^6–8^ In agreement with these studies, a lack of TSLP signaling resulted in reduced Th2 cell-mediated inflammation in murine models of airway inflammation and atopic dermatitis.^9–12^ Based on these findings, an anti-human TSLP antibody (Tezepelumab, AMG 157) was developed and showed significant suppressive effects on airway inflammation and bronchoconstriction in patients with asthma.^13,14^ Therefore, TSLP is increasingly being recognized as a critical cytokine inducing type 2 inflammation and a therapeutic target in the treatment of allergic diseases.

Despite substantial progress in understanding the physiological and pathological roles of TSLP, how TSLP initiates and coordinates type 2 inflammation remains incompletely understood. Various immune cells have been reported to express TSLP receptor (TSLPR), including dendritic cells (DCs), basophils, CD4 + T cells, and group 2 innate lymphoid cells (ILC2s), and disparate cell types have been proposed to have an essential role in TSLP-mediated type 2 inflammation.^15–26^ For example, evidence suggested that TSLP directly stimulated DCs and enhanced the expression of both OX40 ligand (OX40L) and the chemokine CCL17, which in turn contributed to the induction of Th2 cells.^15,16^ However, while the TSLP-DC pathway is often considered to be the most critical TSLP-dependent pathway, other studies indicated that TSLP directly induced the proliferation, survival, and expression of IL-4 in CD4 + T cells^17,18^ and effector Th2 cells.^19^ Furthermore, more recent studies indicated that TSLP directly activated basophils and that basophil-derived IL-4 promoted the induction of Th2 cells and ILC2s,^20–23^ which produce type 2 cytokines in response to IL-33 or IL-25 stimulation. TSLP has also been shown to be capable of enhancing the activation of ILC2s when used as a co-stimulatory cytokine.^24–26^ Thus, given the diverse immune mechanisms described for TSLP, a systematic analysis of cellular targets of TSLP in vivo is needed to parse the relative contribution of each of these cellular pathways in the context of innate versus adaptive immune-mediated type 2 inflammation at mucosal sites.

To this end, we generated multiple cell lineage-specific TSLPR-deficient mice using a Cre/loxP system and dissected the cell-intrinsic requirements for TSLP responsiveness in the context of type 2 airway inflammation. Our data reveal that TSLP regulates distinct immune cell cascades during the development of innate and adaptive immune-mediated type 2 airway inflammation.

## Results

### TSLP plays a vital role in papain-induced innate immune-mediated type 2 airway inflammation

Papain is a proteolytic enzyme that is used in food industries and is known to cause occupational asthma.^27,28^ Mechanistically, papain triggers the release of IL-33 in the lungs, which subsequently activates ILC2s to induce eosinophilic inflammation through the production of type 2 cytokines.^24,29^ Intranasal papain administration for 3 consecutive days is considered as a typical model of innate immune-mediated type 2 airway inflammation since it does not require adaptive immunity.^30,31^ Importantly, although papain can also induce the expression of TSLP in the lungs,^24,32^ the role of TSLP in papain-induced type 2 airway inflammation remains equivocal.^24,29,33^

**Fig. 1:**
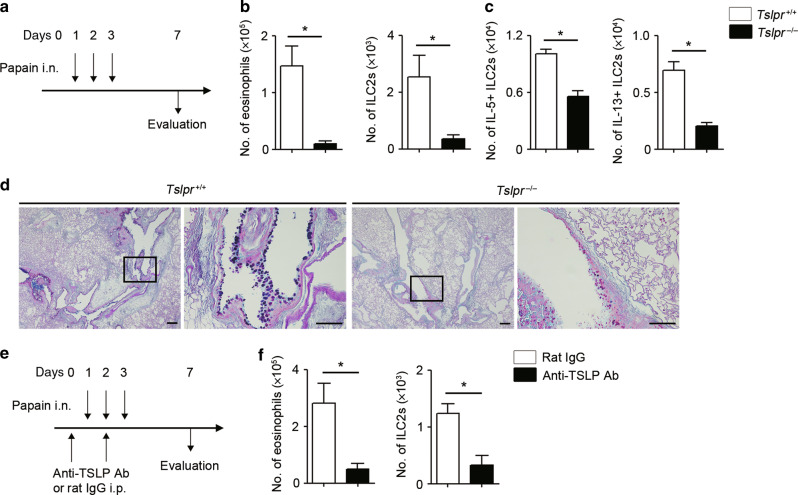
TSLP exacerbates papain-induced type 2 airway inflammation. **a** Experimental protocol for papain-induced airway inflammation model. **b** Number of eosinophils and ILC2s in the BALF (*n* = 4). **c** Number of IL-5- and IL-13-positive ILC2s in the lungs (*n* = 4). **d** Histology of the airways stained with PAS-alcian blue. Scale bars indicate 200 µm (low-magnification) and 100 µm (high-magnification). **e** Experimental protocol for the neutralization of TSLP in vivo. **f** Number of eosinophils and ILC2s in the BALF (*n* = 4). All data are representative of at least 2 independent experiments. *P *<* 0.05 (Mann-Whitney U test).

To test the role of TSLP in an innate immune-mediated type 2 airway inflammation, we first administered papain for 3 consecutive days (on day 1 to 3) and assessed the numbers of eosinophils and ILC2s in the bronchoalveolar lavage fluid (BALF) on day 7 (Fig. 1a, Sup Fig. 1). Eosinophil and ILC2 accumulation was significantly reduced in *Tslpr*^−/−^ mice compared to control mice (*Tslpr*^+/+^) (Fig. 1b). In addition, ILC2s are known to produce type 2 cytokines, including IL-5 and IL-13, and the production of type 2 cytokines from ILC2s was reduced in *Tslpr*^−/−^ mice (Fig. 1c). This reduction of type 2 cytokines in *Tslpr*^−/−^ mice was also accompanied by histological changes. We evaluated goblet cell hyperplasia in the bronchial epithelium of the lung using PAS-alcian blue staining and found dampened goblet cell hyperplasia in the airways of *Tslpr*^−/−^ mice compared to control mice (Fig. 1d). Next, to assess the effect of the neutralization of TSLP, we utilized an anti-TSLP antibody in the papain model. Similar to *Tslpr*^−/−^ mice, treatment of WT mice with an anti-TSLP antibody significantly reduced the accumulation of eosinophils and ILC2s in the papain-induced type 2 airway inflammation (Fig. 1e, f). Collectively, our findings demonstrate that TSLP plays a role in papain-induced innate immune-mediated type 2 airway inflammation.

### TSLP directly affects lymphoid cells, but not basophils

Since our data indicated that TSLP is required for papain-induced type 2 airway inflammation, we next sought to elucidate the immune cell cascades provoked by TSLP. Recent studies have demonstrated that TSLP directly induces basophil hematopoiesis,^22,23,34^ and papain initially activates basophils, which leads to early activation of ILC2s via IL-4.^29^ Indeed, diphtheria toxin (DT)-mediated depletion of basophils in BasTRECK mice significantly suppressed type 2 airway inflammation, as demonstrated by decreased eosinophil and ILC2 numbers (Sup Fig. 2).

**Fig. 2:**
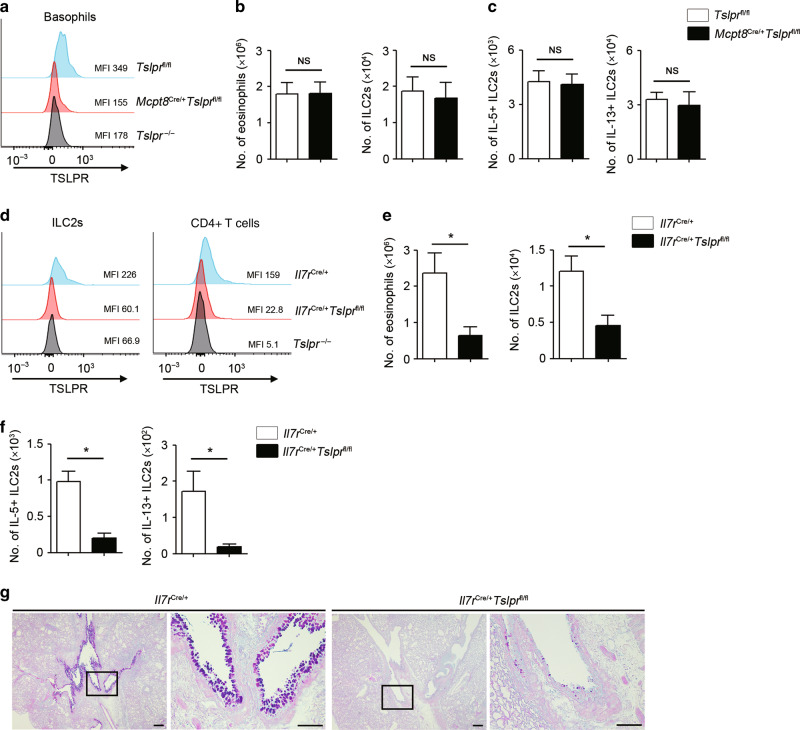
TSLP directly activates lymphoid cells, but not basophils, in papain-induced type 2 airway inflammation. **a** Representative histograms and mean fluorescence intensity (MFI) of TSLPR expression on basophils in different mouse genotypes. **b** Number of eosinophils and ILC2s in the BALF (*n* = 4). **c** Number of IL-5- and IL-13-positive ILC2s in the lungs (*n* = 4). **d** Representative histograms and MFI of TSLPR expression on ILC2s and CD4 + T cells in different mouse genotypes. **e** Number of eosinophils and ILC2s in the BALF (*n* = 4). **f** Number of IL-5- and IL-13-positive ILC2s in the lungs (*n* = 4). **g** Histology of the airways stained with PAS-alcian blue. Scale bars indicate 200 µm (low-magnification) and 100 µm (high-magnification). All data are representative of at least 2 independent experiments. NS; not significant, *P *<* 0.05 (Mann-Whitney U test).

To directly assess whether TSLP-elicited basophils are involved in papain-induced type 2 airway inflammation, we generated basophil-specific TSLPR-deficient mice by crossing *Mcpt8* Cre mice (Basoph8) with *Tslpr*-floxed (*Tslpr*^fl/fl^) mice. As expected, basophils from *Mcpr8*^Cre/+^*Tslpr*^fl/fl^ mice exhibited substantially reduced expression of TSLPR, to the same degree as in *Tslpr*^−/−^ mice (Fig. 2a). However, basophil-restricted deletion of TSLPR had no significant effect on the number of eosinophils and ILC2s following papain administration (Fig. 2b). In addition, IL-5 and IL-13 production from ILC2s was not suppressed in *Mcpr8*^Cre/+^*Tslpr*^fl/fl^ mice (Fig. 2c).

Since basophil-specific deletion of TSLPR failed to influence papain-induced type 2 airway inflammation, we next sought to assess the direct effects of TSLP on lymphoid cells. Lymphoid cell-specific TSLPR-deficient mice were generated by crossing *Il7r* Cre mice and *Tslpr*^fl/fl^ mice. Analysis of TSLPR expression confirmed the loss of TSLPR on lymphoid populations, including ILC2s and CD4 + T cells (Fig. 2d). Intriguingly, when *Il7r*^Cre/+^*Tslpr*^fl/fl^ mice were challenged with papain, type 2 inflammation was significantly suppressed, as indicated by decreased eosinophil and ILC2 numbers, as well as reduced type 2 cytokine production from ILC2s (Fig. 2e, f). Accordingly, *Il7r*^Cre/+^*Tslpr*^fl/fl^ mice also demonstrated significantly reduced goblet cell hyperplasia (Fig. 2g). In addition, *Il7r*^Cre/+^*Tslpr*^fl/fl^ mice showed reduced type 2 airway inflammation compared to control mice even in the absence of CD4 + T cells (Sup Fig. 3), suggesting that other TSLPR-bearing lymphoid cells, presumably ILC2s, are responsible for the effect. Taken together, these data suggest that ILC2s respond to TSLP in papain-induced type 2 airway inflammation, whereas TSLP does not directly affect basophils.

### TSLP directly activates ILC2s to enhance papain-induced innate immune-mediated type 2 airway inflammation

**Fig. 3:**
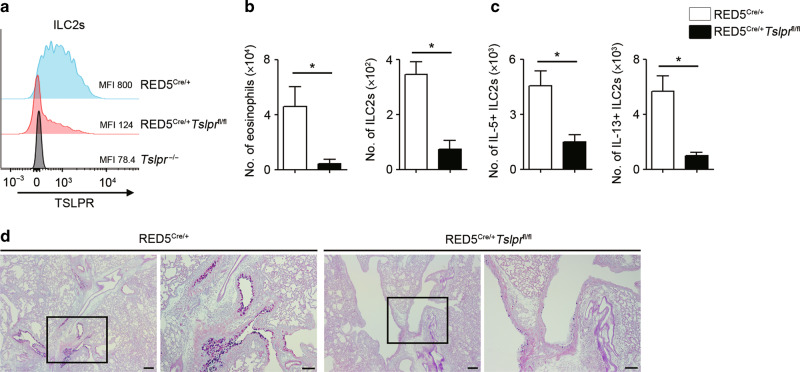
TSLP directly affects ILC2s to enhance papain-induced type 2 airway inflammation. **a** Representative histograms and MFI of TSLPR expression on ILC2s in different mouse genotypes. **b** Number of eosinophils and ILC2s in the BALF of papain-induced airway inflammation model (*n* = 4–5). **c** Number of IL-5- and IL-13-positive ILC2s in the lungs (*n* = 4–5). **d** Histology of the airways stained with PAS-alcian blue. Scale bars indicate 100 µm. All data are representative of at least 2 independent experiments. *P *<* 0.05 (Mann-Whitney U test).

Next, to test whether TSLP directly stimulates ILC2s, we generated mice lacking TSLPR predominantly on ILC2s by crossing RED5 Cre^35,36^ mice with *Tslpr*^fl/fl^ mice. As previously reported, in RED5 Cre mice, Cre is expressed under the control of *Il5* gene, and ILC2s express Cre at steady state.^35^ We confirmed that RED5^Cre/+^*Tslpr*^fl/fl^ mice displayed reduced TSLPR expression on ILC2s (Fig. 3a). In contrast, it is reported that, in these mice, T cells do not express Cre at steady state,^36^ and TSLPR expression on Th2 cells was not significantly reduced on day 7 in the papain model (Sup Fig. 4). Papain-challenged RED5^Cre/+^*Tslpr*^fl/fl^ mice demonstrated a decreased number of eosinophils and ILC2s compared to control mice (Fig. 3b). In addition, type 2 cytokine-producing ILC2s were decreased, and goblet cell hyperplasia was also reduced in RED5^Cre/+^*Tslpr*^fl/fl^ mice (Fig. 3c, d). Although it is reported that IL-33 also plays a critical role in the induction of papain-induced type 2 airway inflammation, IL-33 receptor expression on ILC2s was not affected in RED5^Cre/+^*Tslpr*^fl/fl^ mice (Sup Fig. 5). Therefore, these data demonstrate that TSLP plays an essential role in papain-induced type 2 airway inflammation. In addition, analysis of cell lineage-specific TSLPR deficient mice revealed that TSLP directly stimulates ILC2s in vivo, and enhances type 2 airway inflammation through increased production of type 2 cytokines from ILC2s.

### TSLP plays an essential role in OVA-induced adaptive immune-mediated type 2 airway inflammation

In addition to innate immune-mediated type 2 airway inflammation, it is reported that TSLP plays a critical role in adaptive immune-mediated type 2 airway inflammation, especially in skin-sensitized airway inflammation that models the “atopic march”.^11,12^ While our findings demonstrated that ILC2s directly respond to TSLP in the papain-induced airway inflammation, it has been unclear whether ILC2s are required for TSLP activity during adaptive immune-mediated type 2 airway inflammation.

**Fig. 4:**
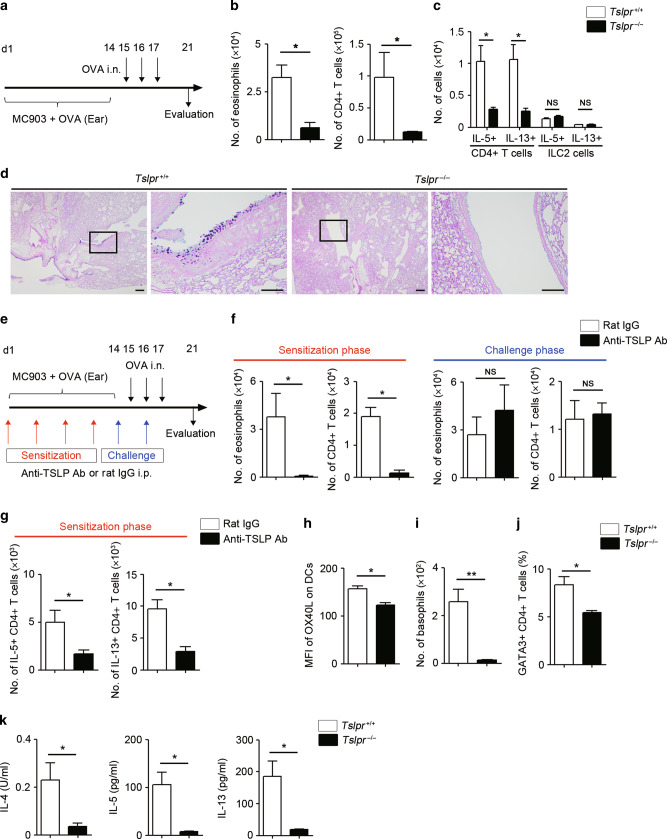
TSLP plays an essential role in OVA-induced type 2 airway inflammation. **a** Experimental protocol for OVA-induced chronic airway inflammation model. **b** Number of eosinophils and CD4 + T cells in the BALF (*n* = 4). **c** Number of IL-5- and IL-13-positive CD4 + T cells and ILC2s in the lungs (*n* = 4). **d** Histology of the airways stained with PAS-alcian blue. Scale bars indicate 200 µm (low-magnification) and 100 µm (high-magnification). **e** Experimental protocol for the neutralization of TSLP in vivo. **f** Number of eosinophils and CD4 + T cells in the BALF following treatment with an anti-TSLP antibody treatment during the sensitization or challenge phase (*n* = 4). **g** Number of IL-5- and IL-13-positive CD4 + T cells following treatment with an anti-TSLP antibody treatment during the sensitization phase (*n* = 4). **h** MFI of OX40L expression on DCs in ear-draining lymph nodes (EDLNs) (*n* = 4–5). **i** Number of basophils in the ear (*n* = 4–5). **j** Percentage of GATA3 + CD4 + T cells in EDLNs (*n* = 4–5). **k** Amounts of IL-4, IL-5, and IL-13 in the culture supernatants of EDLNs following OVA re-stimulation were measured by ELISA (*n* = 4–5). All data are representative of at least 2 independent experiments. NS; not significant, *P *<* 0.05, **P *<* 0.01 (Mann-Whitney U test).

To test this, we utilized the OVA-induced atopic march model.^11,12^ In this model, mice were first sensitized by skin application of OVA with MC903, a vitamin D3 analog that induces atopic dermatitis-like skin inflammation, on the ear for 14 days, followed by intranasal challenge with OVA for 3 consecutive days (Fig. 4a). At day 21, *Tslpr*^−/−^ mice exhibited substantially decreased accumulation of eosinophils and CD4 + T cells (Fig. 4b). Compared to the papain-induced type 2 inflammation model, type 2 cytokines in the OVA model were predominantly produced by CD4 + T cells, rather than ILC2s, and were significantly reduced in *Tslpr*^−/−^ mice compared to control mice (Fig. 4c). In accordance with the decreased production of type 2 cytokine, goblet cell hyperplasia was also diminished in *Tslpr*^−/−^ mice (Fig. 4d).

Next, to assess the temporal effects of TSLP, we utilized an anti-TSLP antibody to therapeutically neutralize TSLP activity during either the sensitization or the challenge phase of allergen-induced inflammation (Fig. 4e). Strikingly, administration of an anti-TSLP antibody during the sensitization phase, but not during the challenge phase, significantly suppressed the accumulation of eosinophils and CD4 + T cells (Fig. 4f). In addition, type 2 cytokine production from CD4 + T cells was significantly reduced after anti-TSLP treatment during the sensitization phase (Fig. 4g).

To further characterize the effects of TSLP during the sensitization phase, we assessed the skin and the skin-draining lymph nodes of *Tslpr*^−/−^ mice. We found that OX40L expression on DCs (Fig. 4h), accumulation of basophils (Fig. 4i), and the percentage of GATA3 + CD4 + T cells (Fig. 4j) were significantly reduced in *Tslpr*^−/−^ mice. Furthermore, type 2 cytokine secretion from ear-draining lymph node (EDLN) cells was markedly reduced in *Tslpr*^−/−^ mice after OVA re-stimulation (Fig. 4k). Collectively, these data indicate that in OVA-induced adaptive immune-mediated type 2 airway inflammation, TSLP plays a critical role in the induction of allergen-specific Th2 cells during the skin sensitization phase, but the role of TSLP is dispensable during the lung challenge phase.

### TSLP directly stimulates DCs, but not basophils, to induce adaptive immune-mediated type 2 airway inflammation

Previous studies demonstrated that both DCs and basophils may be important targets of TSLP for the induction of Th2 differentiation during the sensitization phase.^1,15,16,20–23,34^ TSLP-elicited basophils were suggested to be an essential source of IL-4, which is known to promote the induction of Th2 cells.^20–23,34^ However, results from another study suggested that TSLP-stimulated DCs are capable of inducing Th2 cells from naïve CD4 + T cells via OX40L, even in the absence of IL-4.^16^

**Fig. 5:**
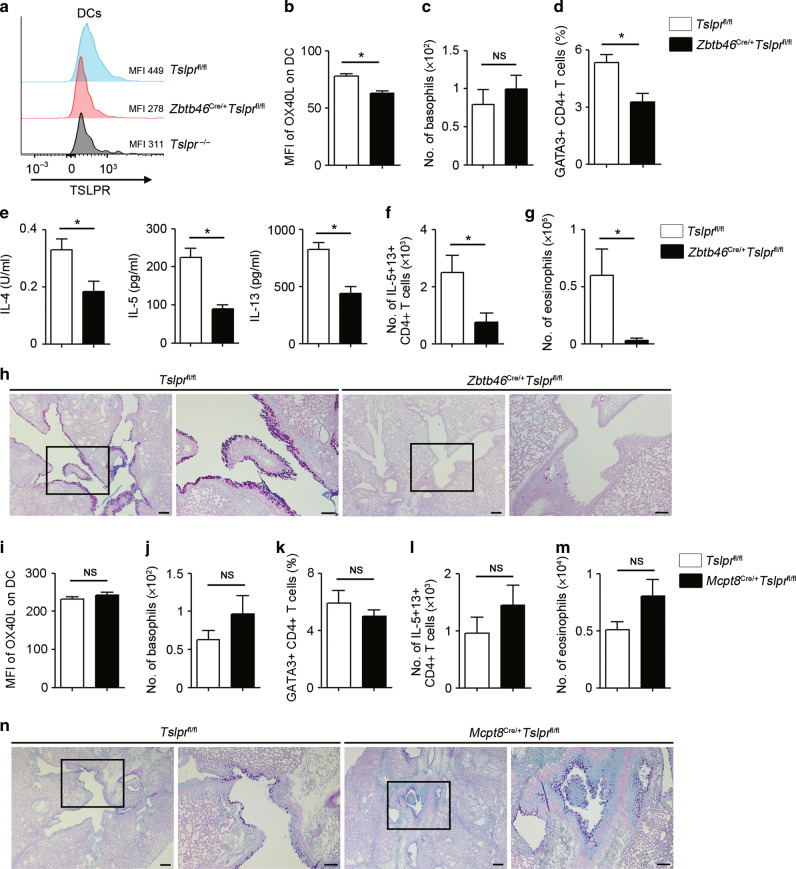
TSLP directly stimulates DCs, but not basophils, in OVA-induced type 2 airway inflammation. **a** Representative histograms and MFI of TSLPR expression on DCs in different mouse genotypes. **b** MFI of OX40L on DCs in ear-draining lymph nodes (EDLNs) (*n* = 3–6). **c** Number of basophils in the ear (*n* = 3–6). **d** Percentage of GATA3 + CD4 + T cells in EDLNs (*n* = 3–6). **e** Amounts of IL-4, IL-5, and IL-13 in the culture supernatants of EDLNs on day 15 following OVA re-stimulation were measured by ELISA (*n* = 3–6). **f** Number of IL-5- and IL-13-positive CD4 + T cells in the lungs (*n* = 4–5). **g** Number of eosinophils in the BALF (*n* = 4–5). **h** Histology of the airways stained with PAS-alcian blue. Scale bar indicates 100 µm. **i** MFI of OX40L on DCs in EDLNs (*n* = 5). **j** Number of basophils in the ear (*n* = 5). **k** Percentage of GATA3 + CD4 + T cells in EDLNs (*n* = 5). **l** IL-5- and IL-13-positive CD4 + T cells in the lungs (*n* = 4–6). **m** Number of eosinophils in the BALF (*n* = 4–6). **n** Histology of the airways stained with PAS-alcian blue. Scale bars indicate 200 µm (low-magnification) and 100 µm (high-magnification). All data are representative of at least 3 independent experiments. NS; not significant, *P *<* 0.05 (Mann-Whitney U test).

To clarify which cell types directly respond to TSLP in vivo, we generated conventional DC-specific TSLPR-deficient mice by crossing *Zbtb46* Cre mice with *Tslpr*^fl/fl^ mice and confirmed that TSLPR expression on DCs was decreased in *Zbtb46*^Cre/+^*Tslpr*^fl/fl^ mice compared to control mice (Fig. 5a). We found that OX40L expression on DCs was reduced in *Zbtb46*^Cre/+^*Tslpr*^fl/fl^ mice following sensitization with OVA + MC903 (Fig. 5b). In addition, GATA3 + CD4 + T cells and type 2 cytokine secretion induced by OVA re-stimulation were both suppressed in *Zbtb46*^Cre/+^*Tslpr*^fl/fl^ mice, whereas the number of basophils was not reduced (Fig. 5c–e). In accordance with these results, intranasal administration of OVA resulted in reduced type 2 airway inflammation as illustrated by reduced number of type 2 cytokine-producing CD4 + T cells, eosinophils, and goblet cell hyperplasia in *Zbtb46*^Cre/+^*Tslpr*^fl/fl^ mice (Fig. 5f–h).

In contrast, compared to control mice, basophil-specific TSLPR deletion in *Mcpt8*^Cre/+^*Tslpr*^fl/fl^ mice showed similar expression levels of OX40L on DCs, similar number of basophils, equal frequencies of GATA3 + CD4 + T cells, and comparable type 2 cytokine secretion induced by OVA re-stimulation (Fig. 5i–k, Sup Fig. 6). Furthermore, there were no differences in type 2 airway inflammation after OVA challenge in *Mcpt8*^Cre/+^*Tslpr*^fl/fl^ mice (Fig. 5l–n). Thus, these findings suggest that TSLP does not directly stimulate basophils in OVA-induced adaptive type 2 airway inflammation, but instead appears to stimulate DCs to promote the induction of allergen-specific Th2 cells and subsequent type 2 airway inflammation.

### TSLP directly stimulates CD4 + T cells, but not ILC2s, to induce adaptive immune-mediated type 2 airway inflammation

**Fig. 6:**
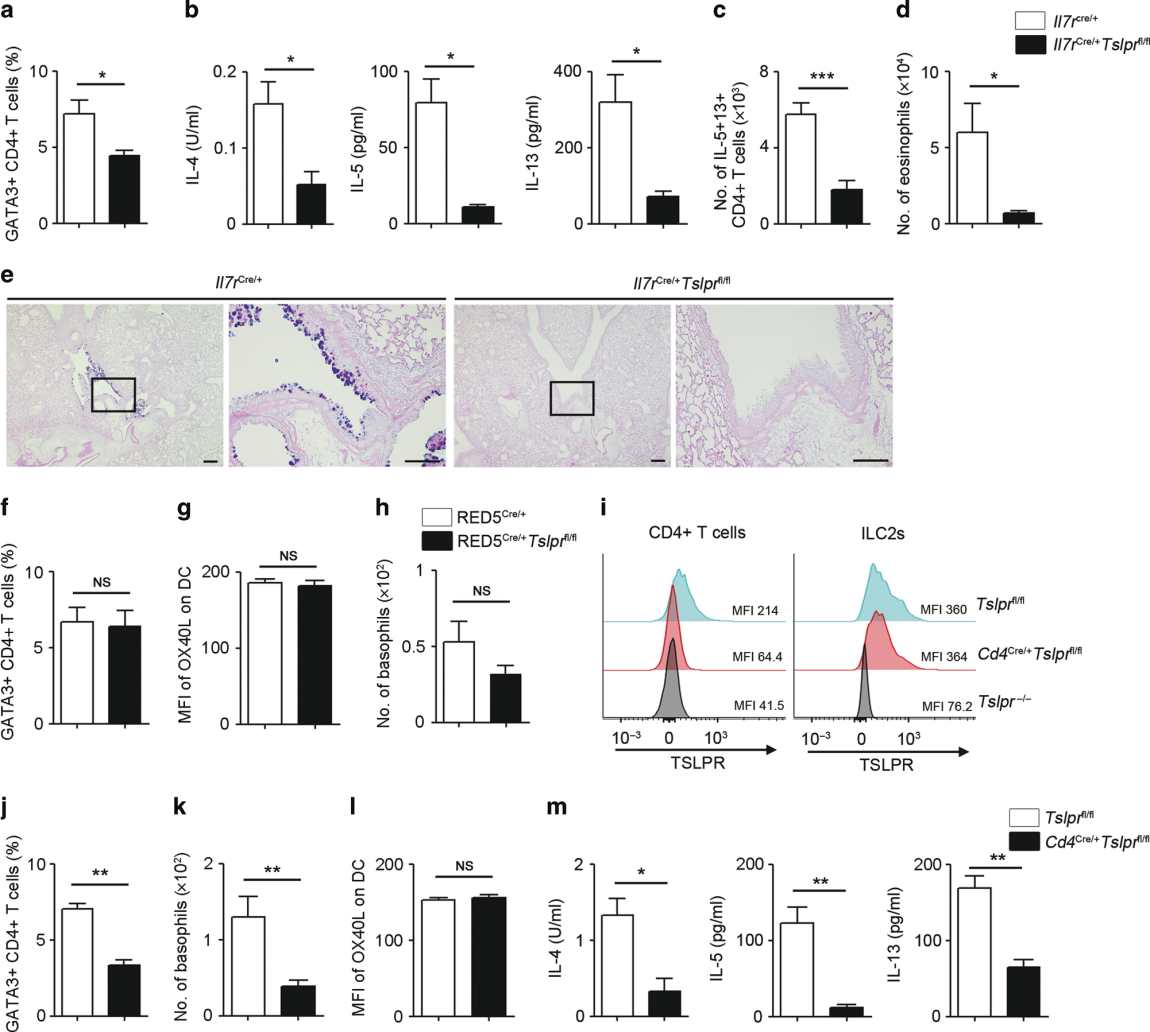
TSLP directly stimulates CD4 + T cells, but not ILC2s, to induce type 2 airway inflammation. **a** Percentage of GATA3 + CD4 + T cells in ear-draining lymph nodes (EDLNs) (*n* = 5). **b** Amounts of IL-4, IL-5, and IL-13 in the culture supernatants of EDLNs following OVA re-stimulation were measured by ELISA (*n* = 5). **c** IL-5- and IL-13-positive CD4 + T cells in the lungs (*n* = 4–5). **d** Number of eosinophils in the BALF (*n* = 4–5). **e** Histology of the airways stained with PAS-alcian blue. Scale bars indicate 200 µm (low-magnification) and 100 µm (high-magnification). **f** Percentage of GATA3 + CD4 + T cells in EDLNs (*n* = 4–5). **g** MFI of OX40L on DCs in EDLNs (*n* = 4–5). **h** Number of basophils in the ear (*n* = 4–5). **i** Representative histograms and MFI of TSLPR expression on CD4 + T cells and ILC2s in the lungs of different mouse genotypes. **j** Percentage of GATA3 + CD4 + T cells in EDLNs (*n* = 4–6). **k** Number of basophils in the ear (*n* = 5–7). **l** MFI of OX40L on DCs in EDLNs (*n* = 5–7). **m** Amounts of IL-4, IL-5, and IL-13 in the supernatants of EDLNs following OVA re-stimulation were measured by ELISA (*n* = 5–7). All data are representative of at least 2 independent experiments. NS; not significant, *P *<* 0.05, **P *<* 0.01, ***P *<* 0.005 (Mann-Whitney U test).

Since our data suggested that TSLP directly stimulates ILC2s to promote papain-induced innate immune-mediated type 2 airway inflammation (Fig. 3), we next evaluated whether TSLP directly affects lymphoid cells during adaptive immune-mediated type 2 airway inflammation. After sensitization with OVA + MC903, the frequency of GATA3 + CD4 + T cells and type 2 cytokine secretion induced by OVA re-stimulation were significantly suppressed in lymphoid cell-specific TSLPR-deficient (*Il7r*^Cre/+^*Tslpr*^fl/fl^) mice (Fig. 6a, b). Furthermore, type 2 airway inflammation and goblet cell hyperplasia after OVA challenge were also reduced in *Il7r*^Cre/+^*Tslpr*^fl/fl^ mice compared to control mice (Fig. 6c–e). Therefore, these data suggest that TSLP directly stimulates lymphoid cells during the sensitization phase to promote subsequent type 2 airway inflammation.

To further dissect the TSLP-related lymphoid cell cascades, we evaluated RED5^Cre/+^*Tslpr*^fl/fl^ mice. In RED5^Cre/+^*Tslpr*^fl/fl^ mice, the number of ILC2s was significantly reduced in the skin during MC903 treatment (Sup Fig 7). However, RED5^Cre/+^*Tslpr*^fl/fl^ mice showed no difference in the frequency of GATA3 + CD4 + T cells, in the expression of OX40L on DCs, and in the number of basophils after the sensitization phase compared to control mice (Fig. 6f–h). Therefore, the TSLP-ILC2 cascade does not appear to be necessary to induce Th2 cells. Next, we generated CD4-specific TSLPR-deficient mice by crossing *Cd4 Cre* with *Tslpr*^fl/fl^ mice. In the *Cd4*^Cre/+^*Tslpr*^fl/fl^ mice, TSLPR expression level was reduced in CD4 + T cells, but not in ILC2s (Fig. 6i). Intriguingly, the frequency of GATA3 + CD4 + T cells, as well as the number of basophils, were significantly suppressed in *Cd4*^Cre/+^*Tslpr*^fl/fl^ mice, although OX40L expression on DCs was not affected (Fig. 6j–l). In addition, OVA re-stimulation-induced type 2 cytokine secretion from EDLN cells was also reduced in *Cd4*^Cre/+^*Tslpr*^fl/fl^ mice (Fig. 6m). Taken together, these findings demonstrate that TSLP directly stimulates not only DCs but also CD4 + T cells during the sensitization phase in the skin. The TSLP-activated CD4 + T cells subsequently promote the induction of basophils and allergen-specific Th2 cells that in turn elicit type 2 airway inflammation. However, TSLP does not directly affect either basophils or ILC2s in the acquired immune-mediated type 2 inflammation. In addition, *Zbtb46*^Cre/+^*Tslpr*^fl/fl^ mice and *Cd4*^Cre/+^*Tslpr*^fl/fl^ mice did not show any reduction of papain-induced innate immune-mediated type 2 airway inflammation (Sup Fig 8). Thus, our study reveals distinct mechanistic effects of TSLP on innate and adaptive immune cells in the context of type 2 airway inflammation.

## Discussion

For more than ten years, TSLP has been described to play an essential role in the induction of type 2 immune response in various diseases, including asthma and atopic dermatitis. Nevertheless, the in vivo TSLP-dependent cascades of cellular responses that promote type 2 inflammation have remained controversial. Here, we defined the in vivo mechanisms through which the innate and adaptive immune system mediate type 2 airway inflammation by utilizing lineage-specific TSLPR-deficient mice.

First, we evaluated the role of TSLP in innate immune-mediated type 2 airway inflammation. Previous studies have shown that papain-induced type 2 airway inflammation is dependent on IL-33, but the role of TSLP in the papain model is equivocal.^24,29,33^ Contrary to expectations, we demonstrated that papain-induced type 2 airway inflammation was significantly reduced in *Tslpr*^−/−^ mice and by anti-TSLP antibody treatment. Furthermore, analysis of lineage-specific TSLPR-deficient mice revealed that TSLP directly affected ILC2s, which enhanced type 2 inflammation in the papain model. In contrast, TSLP-dependent basophil responses did not appear to be required in this model. Moreover, although DCs and CD4 + T cells directly responded to TSLP in the adaptive immune-mediated type 2 inflammation model, we confirmed that both DC-specific and CD4 + T cell-specific TSLPR-deficient mice showed no reduction of inflammation in this model (Sup Fig 8). Therefore, ILC2s are the major responder to TSLP in papain-induced innate immune-mediated type 2 airway inflammation.

Next, we evaluated the role of TSLP in adaptive immune-mediated type 2 airway inflammation and found that the OVA-induced skin-sensitized airway inflammation (atopic march model) was strongly dependent on TSLP. The atopic march refers to the development of atopic dermatitis during infancy and subsequent asthma in later childhood, and the skin is considered to be an important route for allergen sensitization.^37^ Although previous studies suggested that DCs, basophils, CD4 + T cells, and ILC2s were the potential targets of TSLP, there was no systematic analysis.^15–26^ We analyzed lineage-specific TSLPR-deficient mice revealing that both DCs and CD4 + T cells were the major responders to TSLP, and that they enhanced the induction of allergen-specific Th2 cells during the sensitization phase in vivo. In contrast, neither basophils nor ILC2s were involved in type 2 inflammation in this model, although, the reduction of ILC2 accumulation in the skin was dependent on TSLP.

To date, different approaches have been utilized to evaluate TSLP-dependent immune cell cascades in vivo. For example, analysis of TSLPR expression, depletion of TSLPR-expressing cells, and cell transfer experiments to TSLPR-deficient mice have been used to assess the role of the TSLP-TSLPR pathway.^15–26^ However, since the immune system operates through interactive networks, these approaches may lead to controversial results because of the specificity of the depleting antibody or the migration capacity of transferred cells into defined tissues of interest. Indeed, based on these approaches, different research groups reported that distinct cell types, such as DCs, CD4 + T cells, basophils, and ILC2s, directly responded to TSLP and caused type 2 inflammation.^15–26^ Therefore, we used the approach based on the Cre/loxP system to delete the gene encoding the TSLPR specifically in Cre-expressing cell types, allowing the determination of the direct effect of TSLP on a specific cell type in vivo under physiological conditions. Our data, using cell-lineage specific TSLPR-deficient mice, suggest that distinct immune cells directly respond to TSLP and affect local or systemic type 2 inflammation. Although previous cell transfer-based studies suggested that TSLP-stimulated basophils promote type 2 immune responses in the skin following MC903 treatment and in the intestine following helminth infection,^21,22,34^ basophils did not appear to be the primary responders to TSLP in our type 2 airway inflammation models. Furthermore, our data demonstrated that TSLP indirectly induced basophil responses through TSLP-CD4 + T cell pathway. Consistent with this result, other studies also reported that TSLPR on basophils was dispensable for the MC903-elicited allergic skin inflammation based on mixed bone marrow chimera experiments,^38^ and TSLP indirectly induced basophils via CD4 + T cell-derived IL-3.^39^ Together, these data revealed that TSLP does not directly affect basophils in innate and adaptive immune-mediated type 2 airway inflammation. However, it is noteworthy that the present study is focused on the direct effect of TSLP on basophils, and our results are not inconsistent with previous data that basophil-derived IL-4 promotes the induction of type 2 inflammation.^20–23^

Biologics targeting upstream cytokines, such as IL-33 and TSLP, are highly anticipated for allergy treatment. Although IL-33 is a potent inducer of ILC2-mediated type 2 inflammation, it also has a protective role in various diseases, such as myocardial infarction, stroke, and atherosclerosis.^40–42^ Thus, it is possible that an anti-IL-33 antibody may have unfavorable adverse effects for these diseases. On the other hand, TSLP is a cytokine known to promote type 2 inflammation in multiple disease settings, including not only asthma and atopic dermatitis, but also food allergy^22,43^ and eosinophilic esophagitis.^20^ In addition, two clinical studies using an anti-TSLP antibody have shown promising results for asthma without major side effects.^13,14^ Therefore, the use of anti-TSLP antibody is a promising treatment option for allergic diseases by blocking the activation of multiple immune cell cascades as we identified here.

The effect of TSLP on murine type 2 airway inflammation might be variable depending on the type of allergens, time course, and tissues. To further investigate the role of TSLP in adaptive immune-mediated type 2 inflammation models, we intranasally administered papain or house dust mite to mice once a week for three weeks and found that the adaptive immune-mediated type 2 airway inflammation was not suppressed in TSLPR-deficient mice (data not shown). Consistent with these findings, other studies also reported that TSLP was dispensable in house dust mite-induced adaptive immune-mediated type 2 airway inflammation.^44^ Rather, IL-33 was shown to play a critical role in the induction of Th2 cells in the papain- or house dust mite-induced type 2 inflammation.^44,45^ Thus, different allergens or time course may induce the release of different epithelial cell-derived cytokines, which in turn lead to type 2 inflammation via distinct immune cascades. Therefore, in a clinical setting, we may need to assess the effect of TSLP in each asthmatic patient and then specifically select the patients who could have a potential benefit from the therapeutic use of anti-TSLP antibody treatment. In addition, as demonstrated by two different types of murine type 2 airway inflammation models, we cannot entirely rule out the possibility that TSLP plays a role in promoting type 2 inflammation via different immune cell cascades in the context of other stimuli and/or tissues. Therefore, further investigation is required to clarify the TSLP cascades in other mouse inflammation models, as well as patients.

In conclusion, we propose novel in vivo mechanisms through which TSLP-dependent immune cell cascades orchestrate type 2 airway inflammation in the context of innate versus adaptive immunity. Our data demonstrate a critical role for TSLP in innate immune-mediated type 2 airway inflammation, and also confirm the importance of TSLP in adaptive immune-mediated type 2 airway inflammation through skin-sensitization. Furthermore, we show that different TSLP-dependent immune cell cascades induce and orchestrate distinct modules of the immune response. These findings improve our understanding of the complex pathophysiology of allergic inflammation in the lung and emphasize the importance of developing selective treatments targeting upstream cytokines such as TSLP.

## Methods

### Mice

C57BL/6 J mice, *Zbtb46*^cre^ mice on a C57BL/6 background,^46^ and *Cd4*^cre^ mice on a C57BL/6 background^47^ were purchased from the Jackson Laboratory. *Il7r*^cre^ mice on a C57BL/6 background^48^ were provided by Hans-Reimer Rodewald (German Cancer Research Center, Heidelberg, Germany). Basoph8^cre^ mice^49^ and RED5^cre^ mice^35^ on a C57BL/6 background were provided by Richard Locksley (University of California San Francisco, San Francisco, USA). BasTRECK mice on a C57BL/6 background^29^ were provided by Masato Kubo (Tokyo University of Science, Chiba, Tokyo). *Tslpr*^flox^ mice on a C57BL/6 background^43^ were provided by Steven F. Ziegler (Benaroya Research Institute, Seattle, USA). *Tslpr*^−/−^ mice were provided by Amgen.^50^ All mice were maintained in specific-pathogen-free barrier facilities at Weill Cornell Medicine. For all experiments, 6-12-week-old mice were used with age- and sex- matched control mice. All animal experiments and handling procedures were approved by the Weill Cornell Medicine Institutional Animal Care and Use Committees and were performed in accordance with institutional guidelines.

### Reagents and antibodies

Anti-NK1.1 (PK136), anti-CD11c (N418), anti-CD19 (eBio1D3), anti-MHC class 2 (M5/114.15.2), anti-CD49b (HMa2), anti-GATA3 (TWAJ), anti-IL-13 (eBio13A), and anti-CD8 (53-6.7) antibodies were purchased from eBioscience. Anti-Siglec F (E50-2440), anti-CD4 (GK1.5, RM4-5), anti-CD45 (30-F11), and anti-IL-5 (TRFK5) antibodies were purchased from BD Biosciences. Anti-OX40L (RM134L), anti-FcεRI (MAR-1), anti-CD5 (53-7.3), anti-CD3e (145-2C11), anti-CD11b (M1/70), anti-CD127 (A7R34), anti-T1/ST2 (RMST2-33) antibodies, and LIVE/DEAD Fixable Aqua Dead Cell Stain Kit were purchased from ThermoFisher. Anti-CD45 (30-F11), anti-CD16/CD32 (2.4G2), and anti-TSLPR (22H9) antibodies were purchased from Biolegend. Anti-TSLPR (FAB5461P) antibody was purchased from R&D. Anti-TSLP antibody (M702) for in vivo assay was provided by M. Comeau (Aptevo Therapeutics, Seattle, USA).

### Induction and treatment in mouse models

For the induction of inflammation in the papain model, mice were anesthetized with Isothesia isoflurane (Henry Schein Animal Health) and 30 μg of papain (#5125, Calbiochem) in 30 μl of PBS was administered for 3 consecutive days (on day 1, 2, 3), and sacrificed on day 7. For depletion of CD4 + T cells, 250 μg of anti-CD4 antibody (GK1.5, purified in-house) or rat IgG control antibody (I4131, Sigma-Aldrich) was administered intraperitoneally on day 0, 2, 4, and 6. For neutralization of TSLP, mice were treated intraperitoneally with 500 μg of anti-TSLP antibody (M702) or 500 μg of rat IgG control antibody (I4131, Sigma-Aldrich) in 100 μl of PBS intraperitoneally on day 0 and 2. For basophil depletion, BasTRECK mice were administered 500 ng of diphtheria toxin intraperitoneally on day 0 and 3.

For OVA-induced sensitization and challenge model, mice were anesthetized with Isothesia isoflurane and 2 nM of MC903 (Calcipotriol, #2700, Tocris Biosciences) in 20 μl of 100% ethanol was applied to the back of the left ear. After drying, 100 μg of OVA (A5503, Sigma-Aldrich) in 20 μl of PBS was applied to the back of the same ear. As a vehicle control, the same volume of 100% ethanol and PBS were applied. All mice were treated for 14 days for the sensitization phase and then 50 μg of OVA in 40 μl of PBS was administered intranasally on day 15, 16, and 17. For neutralization of TSLP, mice were treated with 500 μg of anti-TSLP antibody or 500 μg of rat IgG control antibody in 100 μl of PBS intraperitoneally on day 0, 4, 8, and 12 for the sensitization phase or on day 14 and 16 for the challenge phase. For the analysis of the sensitization phase, mice were sacrificed on day 15 and ear and EDLNs were analyzed. For the analysis of airway inflammation, mice were sacrificed on day 21.

### Analyses of lung, lymph nodes, and ear tissues

For the analysis of lungs, BALF was obtained by flushing lungs twice with 0.7 ml PBS using a tracheal cannula (Terumo, SURFLO, 20 G). Then, the left lung lobe was fixed in 4% paraformaldehyde (Bioworld) for histological analysis. The right lung lobes were minced and digested with 50 μg/ml of Liberase-TM (Roche) and 20 ng/ml of DNaseI (D5025, Sigma-Aldrich, 750 Kunits/mg) in RPMI 1640 Medium (ThermoFisher) for 1 h at 37 °C, then mashed through 40 μm cell strainers to obtain single cell suspension. For the lysis of red blood cells in BALF and lung single cell suspension, ACK lysing buffer (Lonza) was used. Then, isolated cells were stained and analyzed by FACS.

For the analysis of lymph nodes, EDLN was chopped and digested with 1 mg/ml of Collagenase 2 (C6885, Sigma-Aldrich) and 40 ng/ml of DNaseI in RPMI 1640 Medium for 30 min at 37 °C, then filtered through 70 μm cell strainers. Isolated cells were then analyzed by FACS.

For the analysis of ear tissue, ear was minced and digested with 50 μg/ml of Liberase-TM and 20 ng/ml of DNaseI in RPMI 1640 Medium for 1 hr at 37 °C, then mashed through 40 μm cell strainers to obtain single cell suspension. The isolated cells were subsequently analyzed by FACS.

### FACS analysis

Single cell suspensions from BALF, lungs, ear, and lymph nodes were stained with antibody mixtures and analyzed using a FACS Fortessa (BD Biosciences). Eosinophils were identified as CD45+SiglecF+CD11c− cells, CD4 + T cells were identified as CD45+CD3+CD4+ cells, DCs were identified as CD45+CD64−CD11c+MHCclass2+ cells, basophils were identified as CD45dimFcεRI+CD49b+ cells and ILC2s were identified as CD45+Lin−CD127+T1/ST2+ cells. The lineage markers included CD3, CD5, CD19, NK1.1, CD11c, CD11b, and FcεRI. For intracellular staining of cytokines, cells were stimulated with PMA (30 ng/ml; Sigma-Aldrich), ionomycin (500 ng/ml; Calbiochem), and brefeldin A (10 μg/ml; Sigma-Aldrich) at 37 °C for 3.5 h. Then, cells were fixed and permeabilized using the Fixation/Permeabilization Solution Kit (BD Biosciences). For intracellular staining of GATA3, cells were fixed and permeabilized using the Foxp3/Transcription Factor Staining Buffer Set (eBioscience). All data were analyzed using FlowJo version 10 (TreeStar).

### Ex vivo re-stimulation assay

For antigen re-stimulation assay, 5 × 10^5^ EDLN cells were cultured in 96-well round bottom plates in 200 μl RPMI-1640 media containing 10% FBS, penicillin, streptomycin, and 2-mercaptoethanol with 1 mg/ml OVA at 37 °C for 4 days. Then, 150 μl of supernatants were collected for the cytokine assay.

### Quantification of cytokines by ELISA

Concentrations of IL-4, IL-5, and IL-13 in culture supernatants were measured using ELISA kits (IL4 and IL-5; Biolegend, IL-13; R&D Systems) according to the manufacturer’s protocol.

### Histological analysis

The left lung lobe was inflated and fixed with 4% paraformaldehyde, followed by embedding in paraffin. 5–6 μm sections were used for staining with hematoxylin and eosin or periodic acid-Schiff (PAS) with alcian blue stain by IDEXX BioResearch. Images were acquired under an inverted Nikon Eclipse Ti microscope (Nikon).

### Statistics

All data are presented as mean±standard error unless otherwise indicated. Data were analyzed with GraphPad Prism software (GraphPad). For statistical tests involving two groups, a nonparametric Mann-Whitney U test was used. All *P*-values were based on two-sided tests, and *P*-values less than 0.05 were considered statistically significant.

## Supplementary information

Supplemental Figures
