# Supplementary material for: Targeted deletion of the TSLP receptor reveals cellular mechanisms that promote type 2 airway inflammation
Source: Mucosal Immunol. 2020 Feb 17;13(4):626–36. doi: 10.1038/s41385-020-0266-x (PMC7311324; doi:10.1038/s41385-020-0266-x)
Supplement: Supplementary file 1 — Supplemental Figures [file 41385_2020_266_MOESM1_ESM.doc]

**
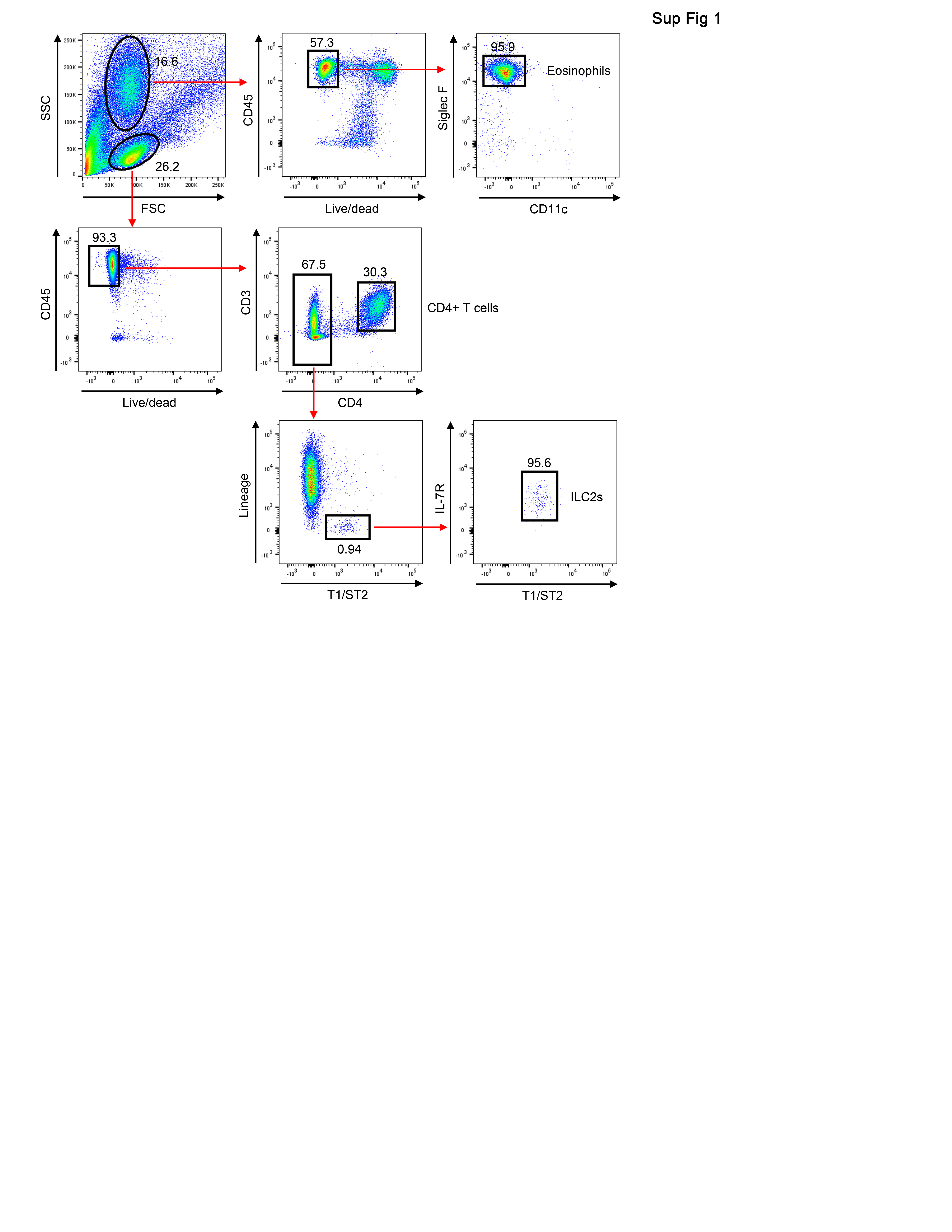
**

**Supplementary figure 1. Flow cytometry gating strategy for eosinophils, CD4+ T cells, and ILC2s.** Eosinophils are identified as CD45+SiglecF+CD11c- cells, CD4+ T cells are CD45+CD3+CD4+ cells, and ILC2s are CD45+Lineage marker-IL-7R+T1/ST2+ cells. Lineage markers include CD3, CD5, CD19, NK1.1, CD11c, CD11b, and FcεRI.

**
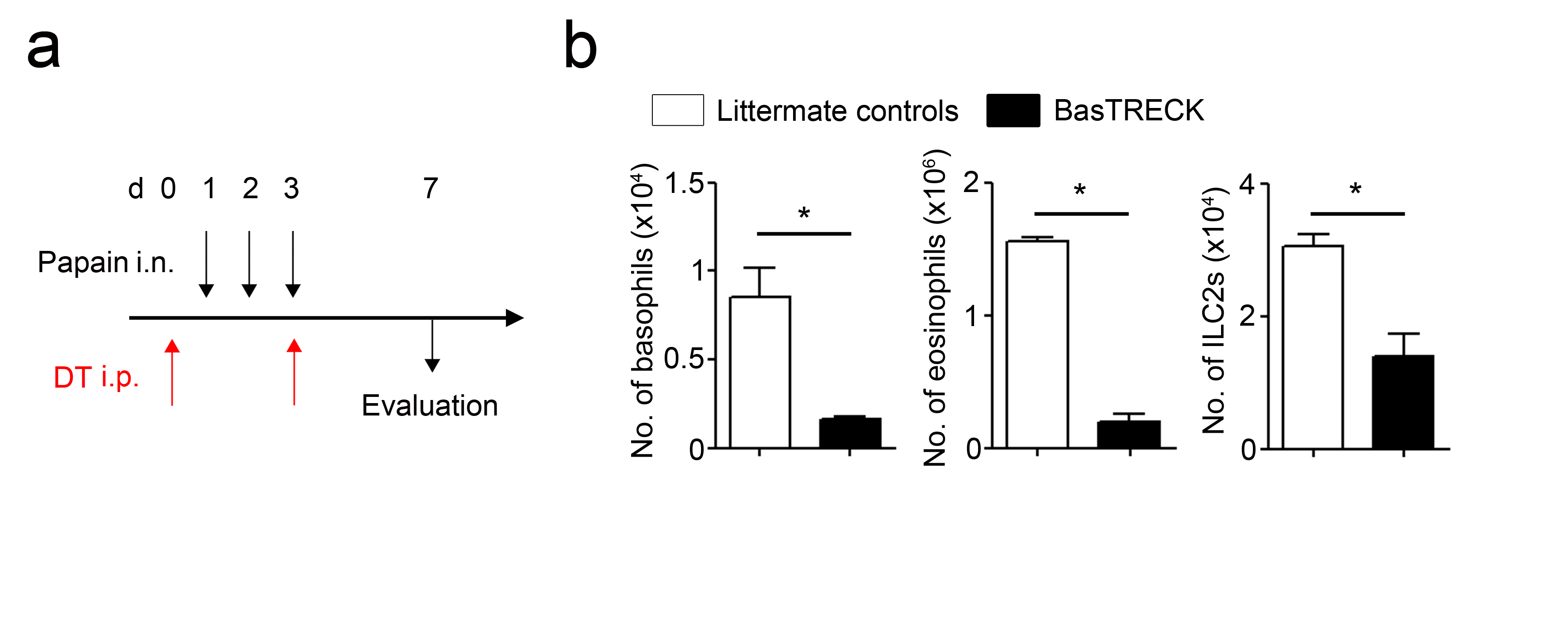
**

**Supplementary figure 2. Papain-induced type 2 airway inflammation in BasTRECK mice**

(**a**) Experimental protocol for papain-induced inflammation model with diphtheria toxin (DT). (**b**) Number of basophils (in the lung), eosinophils, and ILC2s (in the BALF) on day 7. (n = 4) **P* < 0.05.


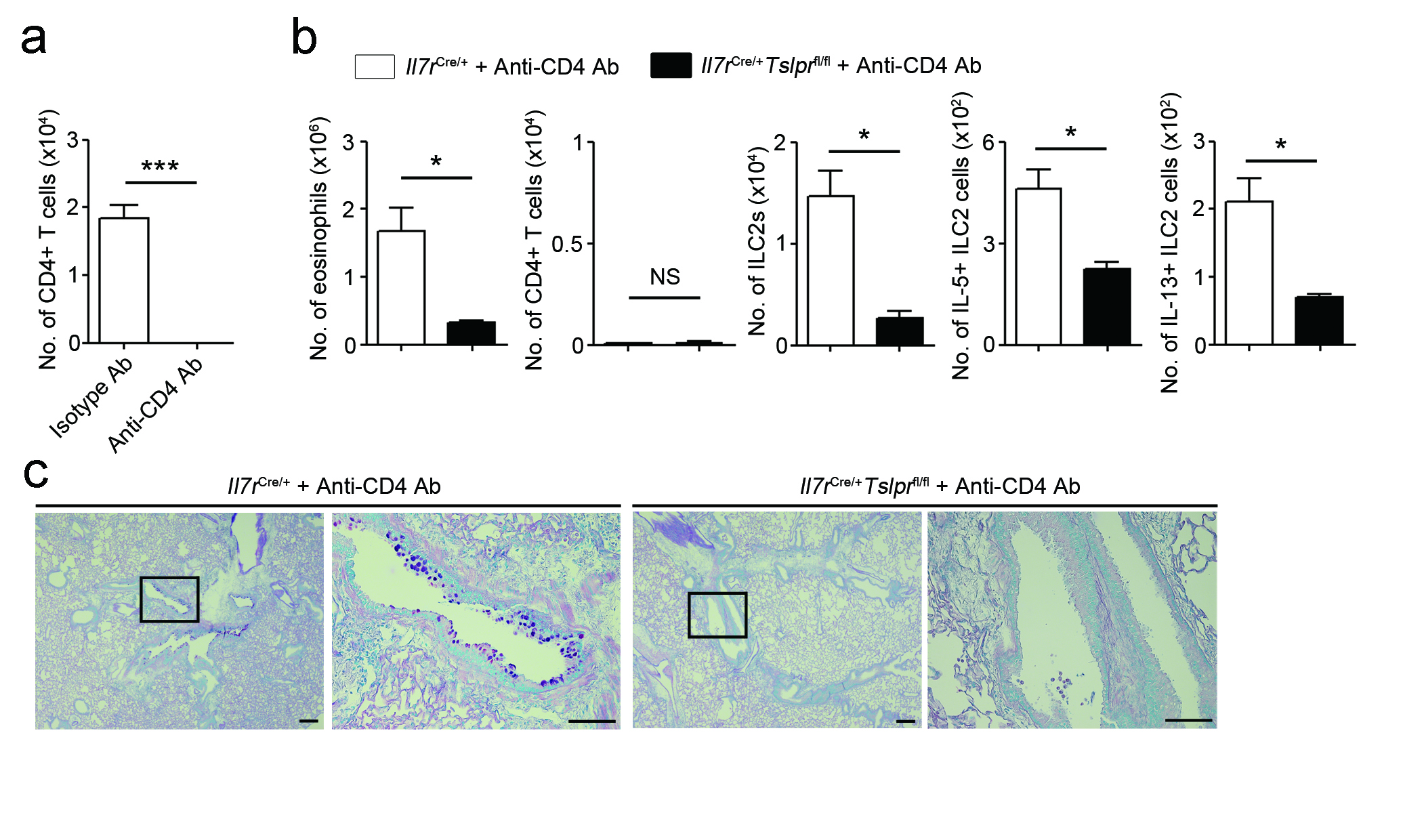


**Supplementary figure 3. Papain-induced type 2 airway inflammation in *Il7r*^Cre/+^*Tslpr*^fl/fl^ mice after treatment with an anti-CD4 depleting antibody**

For depletion of CD4+ T cells, 250 μg of anti-CD4 antibody (Clone: GK1.5) was administered intraperitoneally on day 0, 2, 4, and 6 of the papain model. (**a**) Number of CD4+ T cells (Clone: RM4-5) in the lungs after treatment with an anti-CD4 Ab or an isotype control. (**b**) Number of eosinophils, CD4+ T cells (Clone: RM4-5), and ILC2s in the BALF on day 7. Number of IL-5- and IL-13-positive ILC2s in the lungs on day 7. (n = 3 - 4) NS; not significant, **P* < 0.05, ****P* < 0.001. (**c**) Histology of airways on day 7 stained with PAS-alcian blue. Scale bars indicate 200 µm (low-magnification) and 100 µm (high-magnification).

**
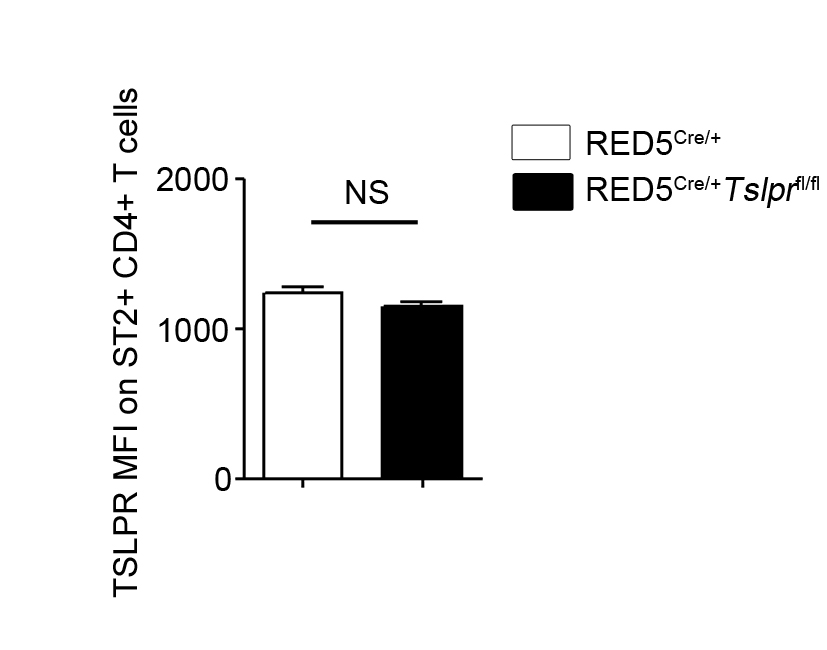
**

**Supplementary figure 4. TSLPR expression on lung Th2 cells in RED5^Cre/+^ and RED5^Cre/+^*Tslpr*^fl/fl^ mice.** Mean fluorescent intensity (MFI) of TSLPR expression on lung T1/ST2+CD4+ T cells in RED5^Cre/+^ and RED5^Cre/+^*Tslpr*^fl/fl^ mice on day 7 of the papain model. (n = 4 - 5) NS; not significant.

**
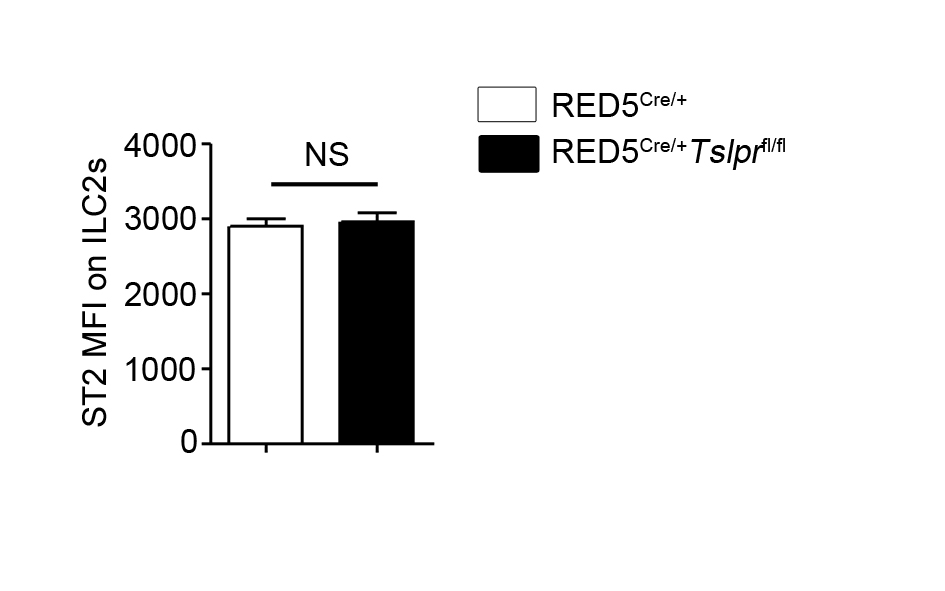
**

**Supplementary figure 5. IL-33R(T1/ST2) expression on lung ILC2s in RED5^Cre/+^ and RED5^Cre/+^*Tslpr*^fl/fl^ mice.** Mean fluorescent intensity (MFI) of T1/ST2 expression on lung ILC2s in RED5^Cre/+^ and RED5^Cre/+^*Tslpr*^fl/fl^ mice on day 7 of the papain model. (n= 4 - 5) NS; not significant.

**
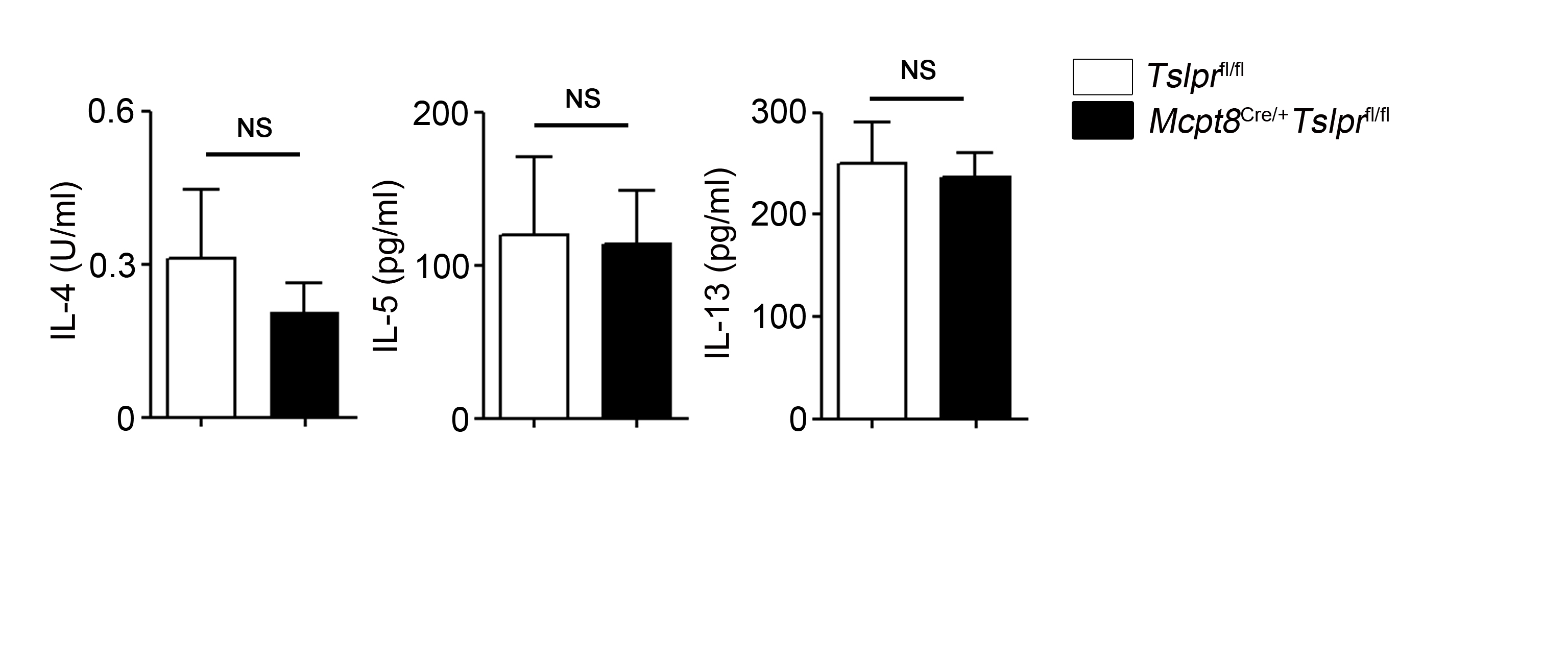
**

**Supplementary figure 6. IL-4, IL-5, and IL-13 secretion in the culture supernatants of EDLNs isolated from *Tslpr*^fl/fl^ and *Mcpr8*^Cre/+^*Tslpr*^fl/fl^ mice.** Amounts of IL-4, IL-5, and IL-13 in the supernatants of EDLNs on day 15 following OVA re-stimulation were measured by ELISA. (n = 6 - 8) NS; not significant.


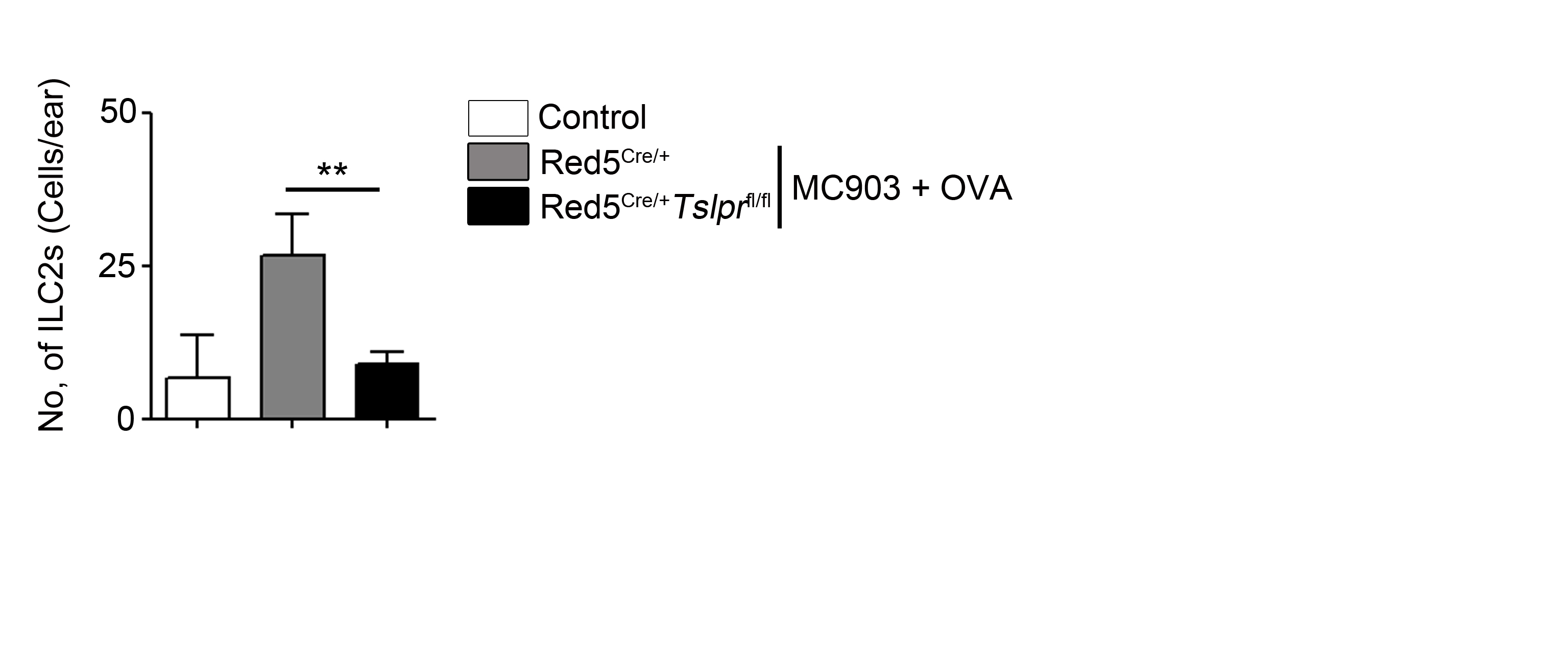


**Supplementary figure 7. Number of ILC2s in the skin after MC903 + OVA treatment.** Number of ILC2s in the ear of RED5^Cre/+^ and RED5^Cre/+^*Tslpr*^fl/fl^ mice on day 15 of the MC903 + OVA model. The control mice were treated by vehicle (EtOH) for 15 days. (n= 4 - 5) ***P* < 0.01.

**
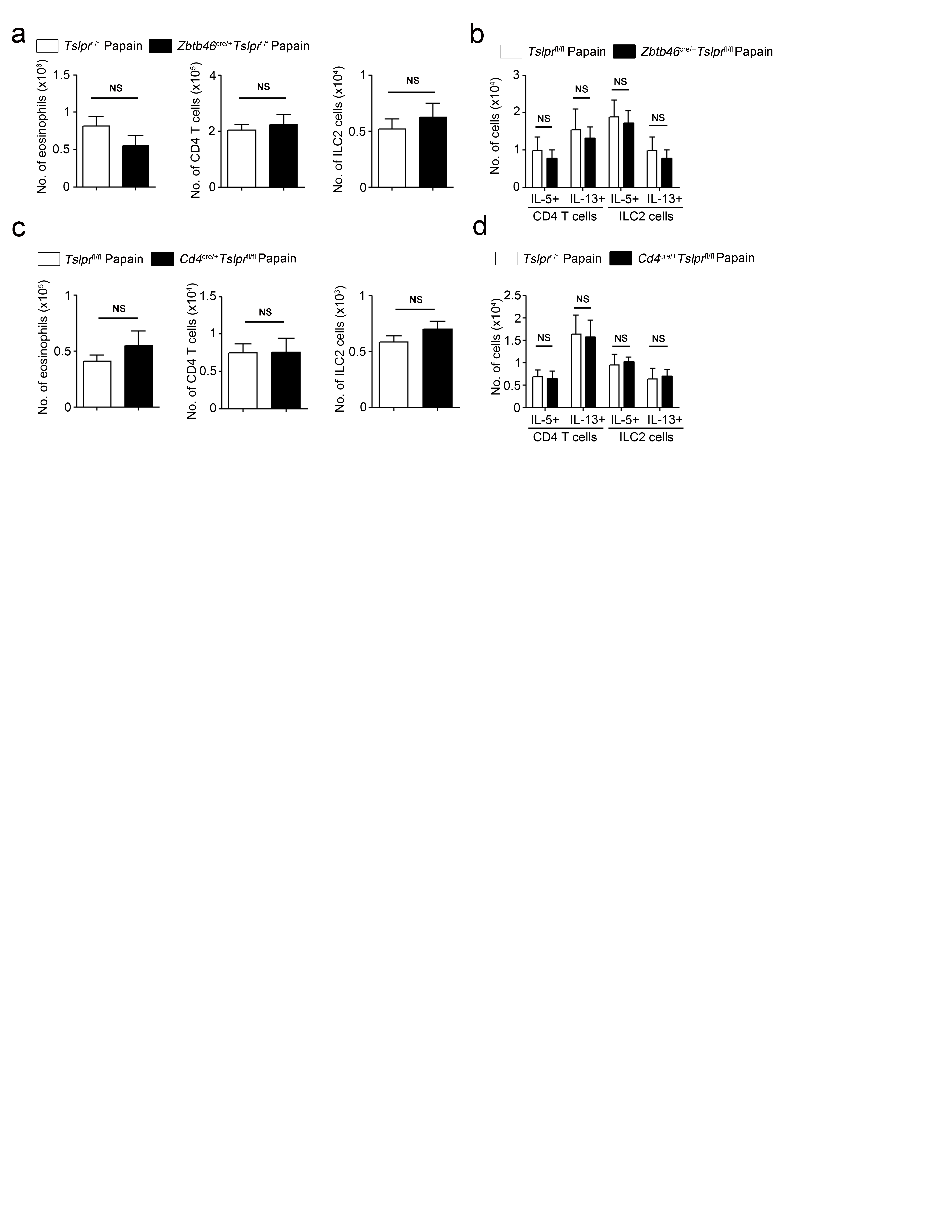
 Supplementary figure 8. Papain-induced type 2 airway inflammation in *Zbtb46*^Cre/+^*Tslpr*^fl/fl^ and *Cd4*^Cre/+^*Tslpr*^fl/fl^ mice**

(a) Number of eosinophils, CD4+ T cells, and ILC2s in the BALF. (b) Number of IL-5- and IL-13-positive CD4+ T cells and ILC2s in the lungs. (c) Number of eosinophils, CD4+ T cells, and ILC2s in the BALF. (d) Number of IL-5- and IL-13-positive CD4+ T cells and ILC2s in the lungs. (n = 4) NS; not significant.
